# The Effects of Gamma-Decalactone on the Physicochemical and Antimicrobial Properties of Pectin-Based Packaging Films

**DOI:** 10.3390/ma18163831

**Published:** 2025-08-15

**Authors:** Gabriela Kozakiewicz, Jolanta Małajowicz, Magdalena Karwacka, Agnieszka Ciurzyńska, Karolina Szulc, Anna Żelazko, Monika Janowicz, Sabina Galus

**Affiliations:** 1 Department of Food Engineering and Process Management, Institute of Food Sciences, Warsaw University of Life Sciences, 159c Nowoursynowska St., 02-776 Warsaw, Poland; s206681@sggw.edu.pl (G.K.); magdalena_karwacka@sggw.edu.pl (M.K.); agnieszka_ciurzynska@sggw.edu.pl (A.C.); karolina_szulc1@sggw.edu.pl (K.S.); s206731@sggw.edu.pl (A.Ż.); monika_janowicz@sggw.edu.pl (M.J.); 2 Department of Chemistry, Institute of Food Sciences, Warsaw University of Life Sciences, 159c Nowoursynowska St., 02-776 Warsaw, Poland

**Keywords:** edible films, active packaging, gamma-decalactone, apple pectin, physicochemical properties, antimicrobial properties

## Abstract

This study introduces an innovative strategy for active, biodegradable food packaging through the incorporation of gamma-decalactone (GDL), a natural aromatic compound with antimicrobial properties, into apple-pectin-based edible films. The addition of GDL significantly modified the film structure, resulting in enhanced light barrier properties (the opacity increased from 1.10 to 8.64 a.u./mm), a more porous microstructure (confirmed by SEM), and reduced tensile strength (from 13.84 to 5.68 MPa). The films also exhibited lower water vapour sorption (from 1.45 to 0.80 g/g dry matter (d.m.) and increased gas permeability. FTIR analysis confirmed interactions between GDL and the polymer matrix. The films with GDL added exhibited antimicrobial properties against various microbial species, such as bacteria, yeasts, and moulds. A 5% addition of GDL to the coating completely inhibited the growth of *Bacillus subtilis* bacteria and *Yarrowia lipolytica*, reducing the number of yeast cells by 3 log units (after 48 h of culture, from 7.11 ± 0.09 to 4.09 ± 0.27 log CFU/mL) and limiting *Monilinia fructicola* mycelium growth by 70%. These results highlight GDL’s dual function as a natural aromatic and antimicrobial agent, supporting its potential application in sustainable packaging for perishable foods.

## 1. Introduction

In the face of the growing problem of environmental pollution with packaging waste and the need to reduce the use of plastics derived from fossil fuels, renewable packaging materials are attracting increasing interest [1]. In the spirit of the circular economy and in line with the assumptions of the European Green Deal, developing innovative, ecological, and functional solutions for food packaging has become one of the key challenges of contemporary scientific and technological research. Among these solutions, biopolymer films play a special role, offering biodegradable and often edible alternatives to conventional plastic packaging [2].

Their advantage, apart from being environmentally friendly, is the possibility of modifying the composition in order to give them specific functions. The functionality of such materials can be increased by introducing active additives, such as essential oils, plant extracts, or chitosan, which give the materials antimicrobial and antioxidant properties, thus helping to protect food from spoilage [3,4]. Aromatic compounds play a special role here: Apart from influencing the sensory properties of products, many of them also exhibit antimicrobial activity [5], and their acquisition by biotechnological methods supports the implementation of the Sustainable Development Goals [6]. Their presence in the packaging structure can perform a dual function: protective and sensory, which increases the attractiveness of the final product without the need to use artificial preservatives.

In recent years, more and more attention has been paid to active and intelligent packaging films, which are able to respond to changes in the environment, e.g., changes in pH and humidity or the presence of certain gases, and, thus, enable the monitoring the freshness and quality of stored food [7,8]. Such solutions include, among others, systems containing natural dyes and indicators, such as extracts from red cabbage, curcumin, or anthocyanins, which can significantly affect the durability and safety of food products [9]. Among natural aromatic compounds, lactones—cyclic chemical compounds with a pleasant, fruity smell, commonly used in the food, pharmaceutical, and cosmetic industries—occupy a special place [10].

One of the most important representatives of this group is gamma-decalactone (GDL), an ester of 4-hydroxydecanoic acid, which is characterised by an intense, peach aroma (C_10_H_18_O_2_) [11,12]. GDL is produced by the β-oxidation of ricinoleic acid, obtained from the hydrolysis of castor oil. Studies have shown that GDL contains about 25 volatile compounds responsible for its characteristic and unique aroma, which makes it highly valued in the food and cosmetic industries [13]. Although GDL occurs naturally in some fruits, its content is so low that obtaining it by extraction methods is economically unprofitable. For this reason, its biotechnological production is preferred, which ensures higher yields and reduced costs [12]. Among microorganisms capable of biosynthesising GDL, the yeast *Yarrowia lipolytica* takes the lead thanks to its high lipolytic activity, which allows the effective conversion of castor oil to GDL [14,15].

GDL is widely used in the so-called white biotechnology, especially in the food, cosmetic, and pharmaceutical industries. Thanks to its intense, fruity aroma, reminiscent of the smell of peaches, this compound is used as an ingredient in fragrance and flavour compositions in products with the aromas of peaches, strawberries, mangoes, apricots, or chocolate, among others [12,15]. The presence of GDL in food and cosmetic product formulations allows us not only to improve sensory qualities but also to obtain a more natural fragrance profile. In addition, GDL has antimicrobial properties, which make it an interesting active additive in modern packaging materials. It can effectively limit the development of undesirable microflora on the surfaces of food products, contributing to their longer shelf lives and improved microbiological safeties [16,17].

One of the effective methods for introducing GDL to packaging materials is microencapsulation—a technique that involves enclosing the active substance within a polymeric shell or matrix. This approach enables the controlled and sustained release of volatile or sensitive bioactive compounds under specific environmental conditions, such as changes in humidity or pH. Common encapsulation materials include polysaccharides, proteins, and lipids. Techniques such as spray-drying, coacervation, and extrusion have proven to be effective in preserving aromatic compounds and enhancing their stability in food-packaging applications [18,19]. For instance, Motelica et al. [20] demonstrated the successful encapsulation of cinnamon essential oil in mesoporous silica nanoparticles and its incorporation into hydroxyethyl-cellulose-based films, resulting in the improved antimicrobial properties and stability of the bioactive compound. Microencapsulation is widely applied in the production of bioactive packaging films, where fragrances, essential oils, antimicrobial agents, or probiotics can be enclosed. Such solutions not only increase the durability and functional properties of the packaging material but also reduce the loss of active substances and improve their stabilities [18,19]. Therefore, the aim of this research was to develop and characterise active packaging films containing GDL regarding their microstructural, optical, mechanical, sorption, barrier, thermal, antioxidant, structural, and antimicrobial properties.

## 2. Materials and Methods

### 2.1. Materials

The research material consisted of edible films made from an apple pectin solution (ZPOW “PEKTOWIN” S.A., Jasło, Poland) with added GDL (Sigma-Aldrich, Poznań, Poland). Glycerol (Avantor Performance Materials Poland, Gliwice, Poland) was used as a plasticiser, while Tween80 (Sigma-Aldrich, Poznań, Poland) acted as an emulsifier. Table S1 presents the compositions of the raw materials used to produce the polysaccharide films. Three species of microorganisms were used in the research on the antimicrobial assessment of the edible films: *Yarrowia lipolytica* yeast KKP 379, from the Collection of Industrial Microorganisms at the Institute of Agricultural and Food Biotechnology in Warsaw (Poland), *Bacillus subtilis* PCM 486, purchased from the Polish Collection of Microorganisms (PCMs) at the Institute of Immunology and Experimental Therapy at the Polish Academy of Sciences (Wrocław, Poland), and *Monilinia fructicola* from the Leibniz Institute DSMZ—German Collection of Microorganisms and Cell Cultures (Braunschweig, Germany). The components of the yeast culture media, including Mueller–Hinton medium and Mueller–Hinton agar, potato dextrose agar (PDA), yeast extract, peptone, and glucose, were purchased from BTL Sp. z o. o. (Łódź, Poland).

### 2.2. Preparation of Film-Forming Solutions and Films

Aqueous film-forming solutions were prepared using apple pectin at a concentration of 5% (5 g/100 g) and heated to 50 °C, maintaining this temperature for 15 min. The solution was stirred with a magnetic stirrer (RCT Basic IKAMAG, IKA Poland Sp. z o.o., Warsaw, Poland) at 500 rpm to achieve a homogeneous consistency. Glycerol was then incorporated as a plasticiser, constituting 30% of the pectin content (1.5 g/100 g). GDL was added in volumes of 2.5, 5, and 10% per 100 g of the solution, alongside Tween 80 (Sigma-Aldrich, Poznań, Poland), used as an emulsifier at 0.5% per 100 g of the film-forming solution. Four films were analysed: AP control films obtained from the apple pectin without the addition of the active compound, and three films with GDL, denoted as AP_2.5GDL, AP_5GDL, and AP_10GDL, corresponding to GD additions of 2.5, 5, and 10%, respectively. The mixtures first underwent preliminary homogenisation using an IKA T25 digital ULTRA TURRAX homogeniser (IKA Poland Sp. z o.o., Warsaw, Poland) at a speed of 10 000 rpm for 2 min, followed by ultrasonic homogenisation with an ultrasonic homogeniser (VC 505, Labo Plus Sp. z o.o., Warsaw, Poland) operating at a power of 20 Hz for 2 min. To achieve a consistent film thickness of 100 ± 10 μm, 50 mL of each film-forming solution was poured into Petri dishes with a diameter of 10 cm. The mixtures were then dried in a laboratory dryer (SMART PRO, POL-EKO APARATURA sp.j, Wodzisław Śląski, Poland) at 50 °C for 24 h, until the films were completely dried. Once dried, the films were carefully removed from the dishes and conditioned in a climate chamber (Binder GmbH, Tuttlingen, Germany) at 25 °C and 50% relative humidity. For each type of mixture, at least three batches of films were prepared to ensure the reproducibility and reliability of the results.

Although drying was conducted at 50 °C for 24 h to ensure complete solvent removal and consistent film formation, we acknowledge that this temperature may influence the polymer network structure. Nevertheless, this condition was selected based on preliminary tests to achieve reproducibility without causing visible thermal degradation of the films. Further research will investigate whether milder drying conditions could achieve comparable film quality without potential structural modifications.

### 2.3. Physicochemical Properties of Pectin Films

#### 2.3.1. Film Thickness

The thickness of the obtained films was measured using a ProGage thickness gauge (Thwing-Albert Instrument Company, West Berlin, NJ, USA) with an accuracy of 1 μm. Measurements were taken randomly from films across different batches, with a minimum of three repetitions. The results were subsequently used to calculate parameters related to mechanical and barrier properties.

#### 2.3.2. Microstructure

The surface structure of the films was examined using a scanning electron microscope (SEM) TM3000 (Hitachi High Tech, Tokyo, Japan). For SEM analysis, 1 × 1 cm specimens of films were cut using operating shears then attached to the measurement table using conductive carbon double-sided discs PELCO (Pik Instruments Sp. z o.o., Piaseczno, Poland) and gold-coated using a sputter coater (108 auto, Cessington Scientific Instruments UK, Watford, UK). Gold is commonly used for coating biological samples due to its electrical and chemical properties [21]. The samples were then placed in the microscope, and the surface and cross-sections of the films were observed using SEM at a magnification of 1500×.

In addition, the microstructure of the films was analysed using a digital optical microscope (VHX-970F, Keyence Corporation of America, Itasca, IL, USA), operating in transmitted light mode, at a higher magnification of 2500×.

#### 2.3.3. Optical Properties

##### UV–Vis Light Transmittance

The UV–vis transmittance of the films was measured using a double-beam UV–vis spectrophotometer (Evolution 220, Thermo Fisher Scientific, Waltham, MA, USA) in accordance with the manufacturer’s manual. The device was equipped with a magnetic film adapter, and measurements were carried out over a wavelength range from 200 to 800 nm. The data were recorded using Thermo INSIGHT software version 2.5 (Thermo Fisher Scientific, Waltham, MA, USA).

##### Colour

Colour was determined using the CIELAB (Lab*) system. A portable colourimeter (Konica Minolta, Tokyo, Japan) was calibrated before testing, using a white reference standard (L* = 97.71, a* = −0.54, b* = 1.20). The colour was measured ten times for each sample, and the average chroma parameters (L*, a*, b*) were calculated. The L* parameter represents lightness and ranges from 0 to 100; the a* parameter indicates the range from green (−120) to red (+120), and the b* parameter denotes the range from blue (−120) to yellow (+120). The total colour difference (Δ*E*) was calculated using the following formula [22]:

$$∆E=\;\sqrt{{\left(L*\;-L\right)}^{2}+{\left(a*-a\right)}^{2}+{\left(b*-b\right)}^{2}}$$

where L*, a*, and b*—the colour parameters of the standard (L* = 97.71, a* = −0.54, and b* = 1.20); *L*, *a*, and *b*—the colour parameters of the tested films.

##### Opacity

The opacity test was carried out using an Evolution 220 UV–visible spectrometer (Thermo Fisher Scientific, Waltham, MA, USA). The tested film samples were placed on a magnetic holder and inserted into the device, which measured the absorbance at a wavelength of 600 nm. Ten repetitions were performed for each type of film. For calculations, the following formula was used [23]:

$$O=\;\frac{A_{600}}{l}$$

where *O*—opacity (a.u./mm); *A*_600_—absorbance at 600 nm; *l*—film thickness (mm).

#### 2.3.4. Mechanical Properties

The mechanical properties, such as the tensile strength and elongation at break, were evaluated using a TA-XT2i texture analyser (Stable Micro Systems, Surrey, UK). The results obtained during the measurements were collected using TextureExpert software (version 2.30). The measurement was performed on strips with dimensions of 25 × 100 mm cut from the tested films. Before the measurements were conducted, the thickness was measured. The films were then placed between two measuring jaws set at a fixed distance of 25 mm, and the measurement was carried out by expanding the jaws at a speed of 1 mm/s. The measurement ended when the sample broke. The mechanical properties were tested in 8 repetitions. To calculate the tensile strength (*TS*), elongation at break (*E*), and Young’s modulus for the initial measurement section (*L*_0_ = 1 mm), the following formulae were used:

$$TS=\;\frac{F_{max}}{A}$$

where *TS*—the tensile strength (MPa); *F_max_*—the maximum force at break (N); *A*—the initial cross-sectional area of the film (mm^2^).

$$E=\frac{∆L}{L}\cdot 100\%$$

where *E*—the relative elongation (%); Δ*L*—the elongation at break (mm); *L*—the initial distance between the grips (mm).

$$M_{Y}=\frac{M_{s}\cdot \Delta L}{A\cdot L_{0}}$$

where *M_Y_*—the Young’s modulus (MPa); *M_s_*—the maximum force (N); Δ*L*—the elongation (mm); *A*—the cross-sectional area (mm^2^).

#### 2.3.5. Barrier Properties

##### Water Vapour Permeability

The water vapour permeability was assessed gravimetrically in accordance with the ASTM E96 standard [24], using a Mater Cup FX-3180 apparatus (Textest AG, Schwerzenbach, Switzerland). Three specimens were cut from each film, and their thickness was measured. The samples were mounted between rubber-sealed rings atop glass vessels filled with distilled water, creating a humidity gradient from 50% to 100%. The instrument calculated the water vapour transmission rate based on the change in the mass over time, the film’s surface area, and the duration of the measurement.

##### Oxygen and Carbon Dioxide Permeabilities

The oxygen permeability was evaluated using a C130 gas permeability tester (Labthink Instruments Co., Ltd., Jinan, China). Film samples were cut to size, and their thicknesses were recorded. Each sample was positioned on a filter within the chamber, and a vacuum was applied. Oxygen was allowed to diffuse through the film into a measuring cell, and the amount of permeated gas was quantified using a manometric technique [25].

#### 2.3.6. Sorption Properties

##### Water Vapour Sorption Kinetics

A saturated sodium chloride (NaCl) solution was used to achieve a constant ambient relative humidity (75%) at 25 °C. The water vapour sorption kinetics for the analysed dry films were measured after 0.5, 1, 3, 6, 9, 12, 24, 48, 72, and 96 h. Samples of films (250 ± 5 mg) were cut into small pieces and weighed periodically. The measurement was carried out at 25 ± 1 °C in three replicates, and the kinetics of the water vapour adsorption were expressed as the amount of water adsorbed per gram of dry matter over time.

##### Water Vapour Sorption Isotherms

The water vapour sorption isotherms of the films were determined using a dynamic vapour sorption analyser, the Aquadyne DVS-2HT (Quantachrome Instruments by Anton Paar Sp. z o.o., Warsaw, Poland). Approximately 20 ± 1 mg of each dry film sample was placed in a glass pan and subjected to a programmed series of relative humidity (RH) levels ranging from 0% to 75% at 25 °C, until equilibrium was attained. The equilibrium at each RH step was considered as achieved when the rate of the mass change (dm/dt) did not exceed 0.002% per minute over a 10 min interval. The measurements were performed in duplicate. Data acquisition and analysis were conducted using Microsoft Excel 2019 in conjunction with DVS Standard Analysis software (latest version Air3). Graphical and statistical processing was carried out in OriginPro 8.0 (OriginLab Corporation, Northampton, MA, USA). The water vapour sorption isotherms of the films were described using the following mathematical models:

− Peleg

$$u=A\cdot a_{w}^{B}+C\cdot a_{w}^{D}$$

− GAB (Guggenheim–Anderson–de Boer)

$$u=\frac{u_{m}\cdot k\cdot c\cdot a_{w}}{\left(1-k\cdot a_{w}\right)\cdot \left[1+\left(c-1\right)\cdot k\cdot a_{w}\right]}$$

where *u* is the water content (g/g d.m.); *u_m_* is the water content in a monolayer (g/g d.m.); *a_w_* is the water’s activity; and *A*, *B*, *C*, *D*, *E*, *F*, *G*, *H*, *c*, and *k* are constants.

The suitability of the models for the description of the sorption isotherms was determined based on the matching of the experimental and predicted data. To select the best equation for describing the sorption isotherms, nonlinear regression analysis in Table Curve 2D v5.01 (Systat Software Inc., San Jose, CA, USA) was used. According to the method described by Ciurzyńska et al. [26], the percentage root-mean-square error (*RMS*), percentage mean relative error (*MRE*), standard error of the estimate (*SEE*), sum of squared residuals (*RSS*), and coefficient of determination (R^2^) fits of each model to the experimental data were assessed from the following equations:

*RMS*—relative-mean-square error (percentage):

$$RMS=\sqrt{\frac{\sum {\left(\frac{u_{e}-u_{P}}{u_{e}}\right)}^{2}}{n}}\cdot 100\%$$

where *u*—the water content (g/g d.m.); data indices: *e*—experimental, *p*—calculated, and *n*—the number of data.

*MRE*—mean relative error

$$MRE=\frac{100}{n}\cdot \sum \left|\frac{u_{e}-u_{p}}{u_{e}}\right|$$

where *u*—the water content (g/g d.m.); data indices: *e*—experimental, *p*—calculated, and *n*—the number of data.

*RSS*—sum of squared residuals

$$RSS=\sum {\left(u_{e}-u_{p}\;\right)}^{2}$$

where *u*—the water content (g/g d.m.); data indices: *e*—experimental and *p*—calculated.

*SEE*—standard error of the estimate for the water content

$$SEE=\sqrt{\sum {\frac{\left(u_{e}-u_{p}\right)}{d_{f}}}^{2}}$$

where *u*—the water content (g/g d.m.); data indices: *e*—experimental, *p*—calculated, and *d_f_*—degrees of freedom (the number of experimental data points minus the number of constants in the model).

#### 2.3.7. Water Contact Angle

The water contact angle of the films was measured via the sessile drop method using a goniometer OCA 25 (DataPhysics Instruments GmbH, Filderstadt, Germany). A 10 μL droplet of distilled water was deposited onto the film surface at a rate of 10 μL/s. Each measurement was repeated three times per film sample. The results were recorded and analysed using SCA20_U software.

#### 2.3.8. Thermal Properties

The thermal stability and decomposition behaviours of the films were analysed via thermogravimetric analysis (TGA), using a Thermal Analysis System TGA/DSC 3+ (Mettler Toledo, Greifensee, Switzerland). Film samples (6 ± 1 mg) were heated from 30 °C to 600 °C at a constant rate of 5 °C/min in a nitrogen atmosphere (flow rate: 50 mL/min). Both the TGA and first-derivative thermogravimetric (dTG) curves were analysed using STARe software (version 19.00).

#### 2.3.9. Antioxidant Properties

##### Free-Radical-Scavenging Activity of ABTS

A solution of 2,2-azino-bis(3-ethylbenzthiozoline-6)-sulphonic acid (ABTS) was prepared from a saturated solution. First, 1 mL was dissolved in 100 mL of ethanol to obtain a solution with an absorbance of between 0.680 and 0.720. Then, the film samples were cut into small pieces (4 × 0.5 cm), put into 5 mL of the diluted ABTS solution, and vortexed. The blank was made with ABTS solution without the film. After 30 min in the dark, the films in the solutions were again vortexed. The absorbance of the samples was then measured with an EVOLUTION 220 UV–visible light spectrometer (Thermo Electron Corporation, Waltham, MA, USA) at 734 nm to determine the antioxidant activity as % inhibition according to the following equation:

$$\%inhibition=\frac{A_{blank}-A_{sample}}{A_{blank}}\cdot 100\%$$

##### Free-Radical-Scavenging Activity of DPPH

A solution of 2,2-diphenyl-1-picrylhydrazyl (DPPH) was prepared from a saturated solution. First, 18 mL of the saturated solution was dissolved in 100 mL of ethanol to obtain a solution with an absorbance of between 0.680 and 0.720. The samples were then cut into small pieces (4 × 0.5 cm) before being put into 5 mL of the diluted ABTS solution and vortexed. The blank was made with a DPPH solution without a film. After 30 min in the dark, the films in the solutions were again vortexed. The absorbance of the samples was then measured with an EVOLUTION 220 UV–visible spectrometer (Thermo Electron Corporation, Waltham, MA, USA) at 515 nm. The antioxidant activity as % inhibition was determined from the equation used for the ABTS calculations.

#### 2.3.10. Fourier-Transform Infrared Spectroscopy

The Fourier-transform infrared (FTIR) spectroscopy of the films was performed using the attenuated total reflectance (ATR) technique and a spectrometer (Cary-630, Agilent Technologies, Santa Clara, CA, USA). The spectra of the analysed materials were recorded in the absorption range from 4000 to 650 cm^–1^ at a resolution of 4 cm^–1^. Each spectrum was obtained as an average of 32 interferograms and presented as absorbance versus wavenumber.

### 2.4. Antimicrobial Properties of the Pectin Films

#### 2.4.1. Inoculation of Microorganisms

Inoculum cultures of *Yarrowia lipolytica* and *Bacillus subtilis* strains were carried out in flat-bottomed flasks in 50 mL of YPG (1% yeast extract, 2% peptone, and 2% glucose) or Mueller–Hinton medium for yeast and bacteria, respectively. The flasks were shaken at 140 rpm at 28 °C (yeast) or 37 °C (bacteria) for 24 h. After this time, 1 mL of the inoculum was transplanted into the appropriate liquid media with biofilms enriched with GDL. The inoculum for the flasks was standardised by measuring the optical density of the culture. An isolate of *Monilinia fructicola* was inoculated on a potato dextrose agar (PDA) medium at 25 °C for 7 days. A sporangiospore suspension was prepared from the flooded 7-day-old cultures and suspended in sterile distilled water. The spore concentration was observed by means of a haemocytometer and adjusted to 1 × 10^5^ spores/mL with sterile distilled water.

#### 2.4.2. Microorganism Cultures

The antimicrobial activities of the edible films, with the addition of GDL, against *B. subtilis* bacteria and *Y. lipolytica* yeast were assessed in batch cultures. Flat-bottomed round flasks were filled with 100 mL of YPG (yeast) or Mueller–Hinton medium (bacteria) and 1 g of edible coating with GDL added at a concentration of 2.5, 5, or 10%. After sterilisation, the media were inoculated with 1 mL of the inoculum. The flasks were incubated at 28 °C (yeast) or 37 °C (bacteria) for 72 h, shaking at 140 rpm. In the case of the *M. fructicola* mycelia, cultures were carried out on 10 cm diameter Petri dishes on a solid PDA medium (100 mL) with 1 g of the coatings with different GDL concentrations, which was introduced before the sterilisation of the agar medium. Cultures were carried out for 7 days at 37 °C. In parallel, control cultures were carried out for all the microorganisms in the media without adding coatings or with a pectin coating without GDL. Each culture was performed in triplicate.

#### 2.4.3. Determination of the Biomasses and Cell Counts

The yeast and bacterial biomasses were characterised based on the cellular dry mass, as measured using the thermogravimetric method. Cells were harvested by centrifugation (at 8000 rpm for 10 min and at 4 °C), washed in distilled water, and dried at 105 °C until reaching a constant weight. Colony-forming units were evaluated based on plate counts. After serial decimal dilutions in physiological saline, the cells were plated onto solid YPD agar (yeast) or Mueller–Hinton agar (bacteria) and incubated at 28 °C and 37 °C, respectively, for 48 h before the cell counting. For each condition, the result corresponded to a mean of three plates and was expressed as log CFU/mL.

#### 2.4.4. Assay of the Effect of the GDL on the Mycelial Growth of *Monilinia fructicola*

*Monilinia fructicola* mycelium was cultured on PDA plates with different concentrations of GDL. The plates without the GDL were used as the blank treatment. Microorganisms were incubated for 3 days at 40 °C. The following formula was applied for calculating the inhibition rate of the *Monilinia*:

$$Inhibition\;rate\;(\%)=\frac{Diameter\;on\;control\;medium\;-\;Diameter\;on\;medium\;with\;GDL}{Diameter\;on\;control\;medium\;}\;\times \;\;100\%$$

### 2.5. Statistical Analysis

The mean values and standard deviations were calculated using Microsoft Excel 2019. Statistical analysis was performed using Statistica 13 software (StatSoft Sp. z o.o., Warsaw, Poland) with one-way analysis of variance (ANOVA) and Tukey’s test. Significant differences between the samples were determined at *p* ≤ 0.05.

## 3. Results and Discussion

### 3.1. Film Characterisation

#### Visual Characteristics of Pectin-Based Packaging Films Containing GDL

**Figure 1:**
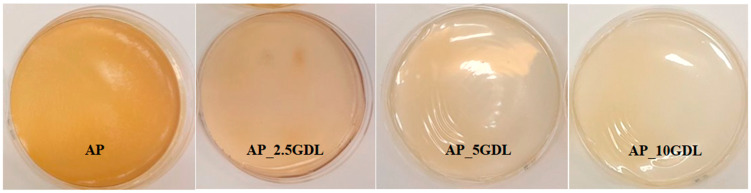
Photographs of apple pectin (AP) films: control and with added GDL.

The films made from the apple pectin, which served as the control sample, were characterised by a smooth, uniform structure with a delicate yellowish hue and slight transparency. After adding the GDL to the film-forming solutions and performing preliminary homogenisation followed by ultrasonic homogenisation, the mixture attained a creamy yellow colour (Figure 1). GDL was added in different amounts (2.5, 5, and 10%) to the film-forming solution, resulting in subtle differences in colour and creaminess. The mixture with the lowest amount of GDL (2.5%) showed a more intense yellow colour but was less creamy. On the other hand, the film with the highest amount of GDL (10%) appeared creamier with a lighter colour. The dried pectin films exhibited a uniform structure and a continuous, reflective surface. No visible cracks or pores were observed. However, for the films containing 10% GDL, some amount of the compound was observed to be released, suggesting that the applied concentration was too high and that the biopolymer matrix was unable to fully incorporate it.

### 3.2. The Effect of the GDL on the Microstructures of the Pectin-Based Packaging Films

**Figure 2:**
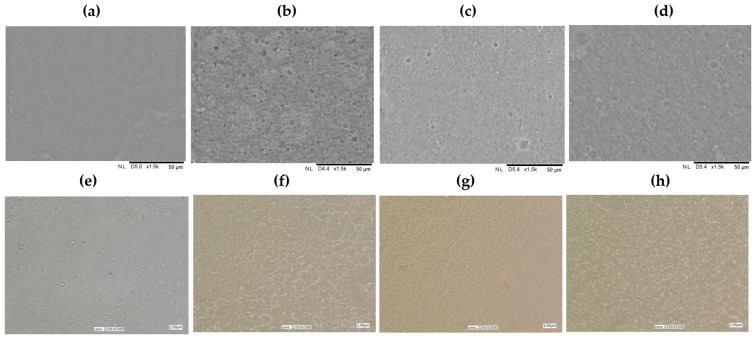
Microscopic images of pectin films (AP) with GDL (GDL). SEM micrographs of corresponding films: (**a**) AP, (**b**) AP_2.5GDL, (**c**) AP_5GDL, and (**d**) AP_10GDL. SEM images were taken at 1500× magnification; scale bar: 50 µm. Light microscopy images of the films: (**e**) AP, (**f**) AP_2.5GDL, (**g**) AP_5GDL, and (**h**) AP_10GDL. Images were taken at 2500× magnification; scale bars: 1 µm.

The structures of the tested control pectin films and those with added GDL were assessed by observing the surface of the materials using scanning electron microscopy. The photos taken at 1500× and 2500× magnifications are shown in Figure 2. The control pectin films exhibited a smooth and homogeneous surface, without any cracks or pores, consistent with typical biopolymer film characteristics [27]. In contrast, the films with added GDL displayed porosity across all the tested concentrations. Notably larger particles were present in some areas, indicated by a darker hue, which suggests the incorporation of the GDL. It is particularly important to note that the films with a concentration of GDL at the level of 2.5% showed a smoother surface, making evaluation easier compared to those of the other formulations. This finding underscores the significance of the additive’s presence and concentration. The analysis indicates substantial structural changes due to adding the GDL, implying that it influences the pectin film’s structure. The variations in the microscopic image assessments may stem from multiple factors, including the interactions between the biopolymer and the active substance and specific production variables, such as the temperature and drying time [28].

Pectin is a complex heteropolysaccharide primarily composed of homogalacturonan, rhamnogalacturonan I, and rhamnogalacturonan II embedded in a covalently bonded structure. Homogalacturonan is negatively charged and consists of galacturonic acid monomers linked by α-1,4 bonds, the carboxyl groups of which are partially esterified. Rhamnogalacturonan I comprises a repeating disaccharide backbone of galacturonic acid and rhamnose, with side chains mainly comprising neutral sugars, such as arabinan or galactose. Rhamnogalacturonan II has a homogalacturonan skeleton with a side chain that comprises four types of structurally complex oligosaccharides [29]. Various interactions can occur between GDL—a cyclic hydroxy acid ester—and pectin, depending on the environmental conditions (pH and temperature). Lactones containing an ester bond in the ring structure can interact hydrophobically with partially hydrophobic fragments of the pectin chain (e.g., methyl groups at galacturonic residues). Pectins contain numerous hydroxyl (–OH) and carboxyl (–COOH) groups and can form hydrogen bonds with the carbonyl group (C=O) in lactone molecules. This can affect the ability to form gel structures, the system’s stability, and the level of lactone binding in the pectin matrix [30].

To fully grasp the effects of the GDL on the biopolymer film structures, further research with more advanced analytical techniques is recommended. The morphologies of the structures of the pectin films with incorporated GDL were observed under transmitted light, and the captured images are presented in Figure 2.

In the control sample (AP), the film’s structure was irregular and non-uniform. Randomly distributed clusters of the material could be observed, which may indicate a lack of homogeneity of the pectin molecule distribution in the film-forming solution. A change in the sample with the addition of 2.5% GDL was visible: The structure became more fine-grained, and the visible elements were smaller and more evenly distributed. This may indicate that the GDL affected the material’s structure, helping the particles to “arrange” better. When GDL was added at 5%, the internal structure looked much more ordered. The structure was more uniform, suggesting that GDL helps to create stronger connections between pectin molecules. Such an effect may be beneficial to the mechanical and barrier properties of the film. At the highest concentration of GDL (10%), the internal structure was densely arranged and more textured. On the one hand, this may mean that the material is even more cross-linked and coherent. Still, on the other hand, it is possible that an excessive amount of the additive caused a high degree of compaction or even cluster formation, resulting in the uniformity of the film’s structure. These findings suggest that GDL may disrupt intermolecular hydrogen bonds within the pectin matrix, leading to a less compact and more flexible structure. Incorporating GDL, a hydrophobic and low-molecular-weight compound, could interfere with pectin–pectin interactions, possibly by inserting between the chains and reducing cohesive forces. This is consistent with similar observations reported in biopolymer systems incorporating lipophilic additives, where adding aromatic compounds or essential oils has been shown to increase the matrix porosity and reduce intermolecular interactions [31,32].

### 3.3. The Effects of the GDL on the Optical Properties of the Pectin-Based Packaging Films

#### 3.3.1. The Effects of the GDL on the UV–Vis Transmission of the Pectin-Based Packaging Films

**Figure 3:**
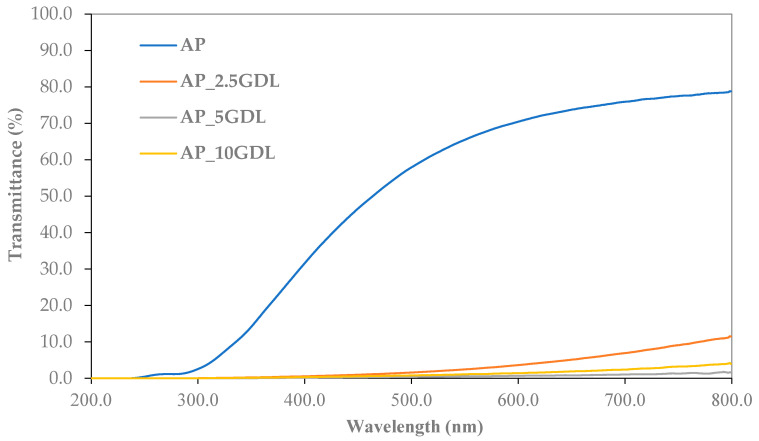
UV–vis light transmittance spectra of the tested apple pectin (AP) films with GDL added.

The data shown in Figure 3 highlight the transmittance values of the edible films derived from the apple pectin (AP), along with their modifications by incorporating GDL at different concentrations. This parameter is a crucial factor that indicates how much light is passed by the film across specific wavelengths. In this study, transmittance measurements were conducted from 200 to 800 nm, facilitating the evaluation of the optical characteristics of the samples in both the ultraviolet and visible light ranges.

The graph analysis indicates that the control film (AP) exhibited the highest transmittance throughout the tested wavelength range, indicating the lowest absorbance. When GDL was introduced, there was a noticeable decrease in transmittance, with this trend becoming more evident as the concentration of the additive rose. The film with a 5% addition of GDL (AP_5GDL) displayed the lowest transmittance values, suggesting it passes the least amount of light in the specified range. However, as shown in Figure 1, the films are not optically transparent, and their surfaces are visibly heterogeneous and partially opaque. The SEM micrographs (Figure 2) also confirm the granular structure, which promotes light scattering. Therefore, the changes observed in the UV–vis spectra should not be interpreted solely as transmittance or absorption. Both diffuse and specular reflection and scattering phenomena contribute to the decrease in transmitted light. Hence, the observed increase in transmittance with the GDL addition likely reflects increased surface roughness and internal scattering caused by GDL precipitation within the pectin matrix rather than a simple increase in molecular absorption. Conversely, the film with a 10% addition of GDL (AP_10GDL) presented a slightly higher transmittance compared to that of AP_5GDL, yet it remained lower than the absorbance of the control film. This observation indicates that enlarging the amount of the GDL did not substantially enhance the light attenuation, which may suggest that above 5% GDL, additional compound does not further alter the film’s scattering or reflection properties, possibly due to the saturation or phase separation of the excess GDL within the pectin matrix.

All the samples exhibited low transmittance in the wavelength range below 300 nm, indicating their capacity to absorb ultraviolet (UV) radiation, primarily in the UV-C and partially in the UV-B ranges (200–300 nm). Botalo et al. [33] also noted similar findings, suggesting that light barrier films can effectively prevent food quality degradation due to photooxidation. Furthermore, other research has demonstrated that cornstarch films containing chitin and chitosan present the maximum absorbance and, thus, the minimum transmittance values within the 250–300 nm range, highlighting their potential in prolonging the shelf lives of products sensitive to photooxidation [34]. Beyond this range, absorbance gradually diminishes, distinguishing materials with UV barrier capabilities while permitting partial light transmission in the UV–vis spectrum. Cai et al. [35] illustrated that in the visible spectrum (350–800 nm), the transparency of edible films stabilised as the wavelength increased. Further analysis of the results, according to López-Ortiz et al. [36], shows that a reduction in the absorbance at longer wavelengths can lead to decreased radiation energy gain in the material, potentially influencing the drying kinetics. Additionally, as highlighted by Ge et al. [22], the transmittance of the visible light is somewhat dependent on the cross-linking of the material, which could clarify the variations observed in samples with different concentrations of GDL.

Incorporating GDL decreased the transmittance of the edible films, thereby altering their optical properties. The most significant decrease in the light transmittance occurs at a concentration of 5%, beyond which this effect becomes stable. The low transmittance in the UV–vis range suggests that these materials may be effective as protective films, especially for light-sensitive products [37]. Simultaneously, controlling the transparency in the visible light spectrum is likely essential for designing films with specific barrier properties.

#### 3.3.2. The Effects of the GDL on the Colour of the Pectin-Based Packaging Films

All the samples were subjected to colourimetric analysis to determine colour parameters such as the lightness (L*) and chromaticity coordinates from green (negative values) to red (positive values) (a*) and from blue (negative values) to yellow (positive values) (b*) [38]. Additionally, the total colour difference (Δ*E*) was calculated relative to a white standard, allowing for an evaluation of the films’ colour changes. At the same time, an opacity analysis of the films was conducted, revealing differences between individual samples. The results of the colour and opacity analyses are presented in Table 1. Films produced with the apple pectin and the addition of the GDL differed in colour. The sample containing 5% GDL (AP_5GDL) stood out the most visually, exhibiting a higher colour difference than those of the other samples.

According to a study by Salem et al. [39], higher L* values indicate a brighter sample appearance, while lower values suggest a darker one. The L* parameter, indicating lightness, reached its highest value in the control sample (83.68), indicating that this film was the brightest among all the tested samples. Samples with GDL showed significantly lower L* values, suggesting that the additive caused the films to darken. The film with 5% GDL had the lowest lightness (74.47), possibly due to a reaction between the GDL and pectin, resulting in colour changes in the material. However, no difference in the L* parameter was recorded between samples AP_2.5GDL and AP_10GDL.

The a* value for the control film was −0.31, indicating a slightly greenish hue. Films with GDL had higher a* values (5.06–6.32), pointing to a tendency toward reddish colours. The differences were the most noticeable in the samples with 5% and 10% of the additive, which showed more intense red shades.

The b* parameter was also affected by the addition of the GDL. The control sample was characterised by a value of 20.91, while samples with this compound featured results in the range 28.64–32.11, with the 5% concentration (AP_5GDL) showing the most saturated yellow tone. This indicates that all the samples exhibited a yellowish colour. Statistical analysis showed a significant dependence (*p* ≤ 0.05) of the b* parameter on the formulation of the samples. According to a study by Ngo et al. [40] on pectin-based edible films, an increase in a* reflects a shift from green to red tones as the value moves from negative to positive. Moreover, an increase in b* indicates a more yellow film appearance.

The colour differences were particularly evident in the total colour difference values (Δ*E*). This parameter can be interpreted as follows: A value below 1 indicates no perceptible change in colour; a trained observer can notice values between 1 and 2. Values between 2 and 3.5 are visible to the untrained eye, and values above 5 suggest a significant colour difference [41]. For the control film, the Δ*E* value was 21.29, indicating a significant difference in colour compared to that of the white reference. The films with GDL (AP_2.5GDL, AP_5GDL, and AP_10GDL) had even higher Δ*E* values of 32.15, 36.07, and 34.05, respectively. According to the accepted classification, Δ*E* values above 5 indicate noticeable colour changes, even by non-specialists, which indicates that the differences between the colours of the tested samples, especially the control and those with the GDL addition, could be clearly visible to even an untrained observer. Statistical analysis confirmed significant differences (*p* ≤ 0.05) in lightness (L*) between the control film and the films containing GDL, indicating that the additive influences the darkening of the films. Similarly, significant differences (*p* ≤ 0.05) were observed in a* and b* values, where the samples with GDL showed more intense red and yellow tones compared to those of the apple pectin film. The total colour difference (Δ*E*) for all the GDL films exceeded 5, confirming clear differences (*p* ≤ 0.05) in colour relative to that of the white reference. The greatest statistical difference (*p* ≤ 0.05) was observed between the control film and the film containing 5% GDL, across all the colour parameters. Similar results were reported by Łopusiewicz et al. [42], who demonstrated that the modification of BOPP and PET films with coatings containing fungal melanins leads to a decrease in the L* value, indicating a darkening of the films. At the same time, increases in the a* and b* values were observed, reflecting a colour shift towards red and yellow hues. High Δ*E* values confirmed significant colour changes compared to the colour of the control samples.

#### 3.3.3. The Effects of the GDL on the Opacity of the Pectin-Based Packaging Films

Although opacity is sometimes misinterpreted as the inverse of the transparency, the two describe different physical phenomena. Transparency refers to the ability of a material to transmit light, whereas opacity describes the extent to which a material prevents light transmission, including both absorption and scattering contributions. However, it does not account for light reflectance, which can also be significant in the case of semi-opaque films [27]. The opacity values presented in Table 1 refer to the degree of the blocking of light rays at a wavelength of 600 nm, which falls within the visible spectrum. In this context, opacity is calculated as the absorbance divided by the film thickness and corresponds to the absorption coefficient (A/l) in accordance with the Lambert–Beer law. It should be noted that this parameter does not include reflection phenomena, which may also significantly affect the optical behaviour [43].

Although the opacity value for AP_10GDL is numerically the highest, this was calculated from a single-point absorbance measurement at 600 nm. In contrast, the spectral data in Figure 3 indicate that AP_5GDL exhibits the highest absorbance across the broader UV–vis range. This discrepancy may result from local heterogeneity or the uneven distribution of the GDL, which can influence different regions of the spectrum differently. Additionally, opacity was calculated at a single wavelength, while UV–vis spectra represent a continuous range of wavelengths. Therefore, localised sample variability and wavelength-dependent scattering may contribute to the apparent inconsistency.

The lowest opacity value (1.10 a.u./mm) was recorded for the control film. In contrast, the materials containing GDL showed significantly (*p* < 0.05) higher values, ranging from 6.47 to 8.64 a.u./mm. The highest opacity value (8.64 a.u./mm) was observed in the film with the highest concentration of GDL (AP_10GDL), making it the most opaque sample among those tested. Films with 2.5% (AP_2.5GDL) and 5% (AP_5GDL) GDL also exhibited increased opacity compared to that of the pure apple pectin film, with values of 6.47 and 7.73 a.u./mm, respectively. These results suggest that the addition of the GDL increased the films’ opacity, thereby enhancing their ability to act as light barriers and indicating that while consumers often prefer more transparent packaging materials, low transparency does not necessarily indicate poor quality or functionality. According to Singh et al. [44], films with higher opacity may be more effective in protecting food from photooxidation, which highlights the potential importance of opacity in preventing degradation.

### 3.4. Effects of the GDL on the Mechanical Properties of the Pectin-Based Packaging Films

Pectin films are materials derived from polysaccharides and find applications in diverse areas, including biopolymer food packaging, biomaterials, and biodegradable materials. The mechanical properties of these films can differ significantly based on the type of raw material used, the production process, the environmental conditions, and any subsequent chemical or physical modifications [45]. In many applications, the mechanical strength and flexibility of pectin films are crucial, especially in situations where high resistance to deformation or plasticity is required. The research methodology focused on assessing the mechanical strengths of the pectin films by evaluating three key parameters: the tensile strength, elongation at break, and Young’s modulus. The findings of this research are detailed in Table 2.

Tensile strength, defined as the ultimate strength or stress at break, is a mechanical parameter describing the maximum tensile stress that a material can withstand [46]. In the tests conducted, the tensile strength values of the films ranged from 4.31 to 13.84 MPa (Table 2). The control film demonstrated the highest tensile strength, while the incorporation of GDL into the polymer matrix resulted in a statistically significant (*p* ≤ 0.05) reduction in the tensile strength, which decreased to the range 4.31–5.98 MPa. This reduction in the tensile strength of the active films suggests a loosening of the structure and increased flexibility due to the added substance, which could be attributed to the separation of some GDL from the film, leading to a loss of the plasticising effect.

This interpretation is further supported by the SEM images (Figure 2) showing increased porosity and surface irregularities in the films containing GDL. The reduced stiffness and compactness of the polymer matrix observed visually are consistent with the observed drops in the tensile strength and Young’s modulus. Furthermore, FTIR analysis (Section 3.11) indicated modifications in peak intensities, suggesting weakened hydrogen bonding and altered interactions between pectin chains due to the presence of the GDL, which may explain the mechanical relaxation observed.

Elongation at break is the measure of how much a material can stretch or change in length relative to its original size when external forces are applied [47]. In the case of the pectin films, the elongation at break varied between 11.50% and 21.26% (Table 2). The control film had a value of 14.00%. When GDL was added, there was a minor initial reduction to 11.50% for the film containing 2.5% of this compound, but this increased to 21.26% for the film with 5%. At the highest concentration (10 mL/100 mL), it decreased again to 19.97%. It is important to note that there were statistically significant differences (*p* ≤ 0.05) in elongation when comparing these two higher concentrations with the control sample.

The Young’s modulus, which is a key indicator of a material’s stiffness, was also evaluated [47]. For the pectin films tested, the Young’s modulus ranged from 108.34 MPa for the control films to between 44.91 MPa and 50.87 MPa for films that included GDL (Table 2). The apple pectin control films showed a stiffness of more than double that of the films with GDL additives, suggesting that this impressive stiffness can be beneficial in applications that demand high resistance to deformation, thus ensuring durability and stable mechanics. The films with the added GDL consistently demonstrated significantly lower Young’s modulus values (*p* ≤ 0.05), though these values appeared similar across varying concentrations. Remarkably, there was an initial rise in the Young’s modulus with the addition of concentrations of GDL (2.5 and 5%) before a decline was noted at the highest concentration. These findings imply that there are interactions among the components of the polymer matrix, factoring in the glycerol plasticiser and the emulsifier (Tween80), which also affected the structures and interactions of the resulting edible films. Similar trends have also been identified in biopolymer films containing aromatic substances. Notably, the film with the 5% GDL exhibited a favourable combination of reduced stiffness and increased elongation at break, suggesting the optimal balance between flexibility and structural integrity. This trade-off is particularly relevant for packaging applications, where moderate extensibility and mechanical resistance are desired, and is consistent with observations in other studies on biopolymer films modified with aromatic compounds [48].

### 3.5. The Effects of the GDL on the Barrier Properties of the Pectin-Based Packaging Films

The barrier properties of edible films refer to the ability of the material to limit the penetration of external factors, such as gases (e.g., oxygen and carbon dioxide), water vapour, or light. Due to these properties, films protect food products against oxidation, loss of moisture, and other negative processes, which affect their durability and quality during storage [49]. Table 3 presents the results of the analysis of the barrier properties of the edible films obtained from apple pectin (AP) with the addition of the GDL at concentrations of 2.5, 5, and 10. The permeabilities of the water vapour, oxygen, and carbon dioxide were assessed. The water vapour permeability (WVP) for the control film (AP) was 1.97 × 10^−11^ g/m·s·Pa. The addition of the GDL caused an increase in this parameter in a concentration-dependent manner: The values were 2.49 × 10^−11^, 2.89 × 10^−11^, and 3.23 × 10^−11^ g/m·s·Pa for the films containing 2.5%, 5%, and 10% GDL, respectively. A statistically significant increase (*p* ≤ 0.05) compared to the control sample was observed only at the highest concentration of GDL, which may indicate a deterioration of the moisture barrier properties.

An analogous trend was observed in the case of the gas permeability. The oxygen permeability for the apple pectin film was 1.49 × 10^−16^ g/m·s·Pa, while those for the films with the added GDL, were 5.65 × 10^−16^ (2.5%), 12.06 × 10^−16^ (5%), and 15.19 × 10^−16^ g/m·s·Pa (10%). This increase was statistically significant for the highest concentration of GDL, which indicates the significantly increased oxygen permeability of the material. Similar relationships were observed for CO_2_ permeability, which increased from 4.47 × 10^−16^ g/m·s·Pa (for the control sample) to 12.74 × 10^−16^ g/m·s·Pa at 10% GDL. Also, in this case, the highest concentration resulted in a significant change compared to the control film. Based on the obtained results, it can be concluded that the film made exclusively of the apple pectin was characterised by the lowest permeabilities for both oxygen and carbon dioxide, which confirms its particularly favourable barrier properties compared to those of composites containing GDL.

Pectin, as a polysaccharide with the ability to form dense and compact polymer networks, effectively limits the penetration of gases, such as oxygen and carbon dioxide, which are the main factors accelerating the oxidation and spoilage processes of food. Additionally, limiting the permeability of the water vapour prevents the loss of moisture from products, which is important for maintaining their textures and sensory qualities [50].

The increases in the permeabilities of both the water vapour and gases may be related to the disruption of the polymer structure of the film due to the addition of the GDL. As a lipophilic and aromatic compound, GDL may affect the cross-linking and organisation of pectin chains, leading to the formation of a less compact and more porous structure [51]. Similar phenomena have been observed in other studies on active edible films, where the addition of bioactive substances led to the deterioration of the barrier properties [52]. The authors indicated that bioactive compounds can disrupt the structure of the polymer network, increasing the material’s permeabilities of gases and water vapour. This effect may also apply to GDL, which, as a lipophilic compound, can affect the organisation of the film structure [51,52].

### 3.6. The Effect of the GDL on the Water Vapour Sorption Kinetics of Pectin-Based Packaging Films

**Figure 4:**
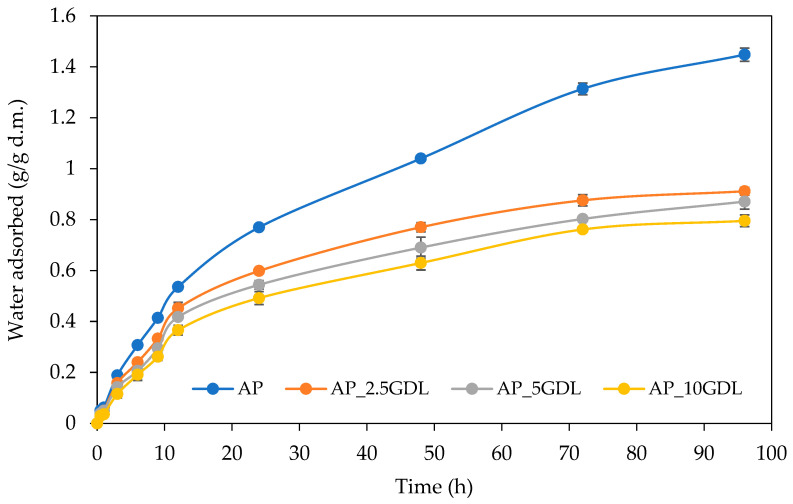
Water vapour kinetic curves of the apple pectin (AP) films containing GDL.

The water vapour sorption properties of the studied pectin films with and without the addition of the GDL were analysed by determining the kinetic curves that explore the dependence of the change in the amount of absorbed water vapour over time. The obtained kinetic curves are presented in Figure 4. In general, the water vapour sorption kinetics depend on the sorption time, environmental relative humidity, and sample type. In the analysis of the presented results, the shapes of the kinetic curves and the values of the water adsorption have shown the apparent effects of the GDL addition on the water vapour sorption rate. The control films showed a higher adsorption capacity in comparison to the films containing aromatic compounds, indicating that the composite films absorb a lower amount of water vapour over 96 h of the process. This difference was observed at all times. However, the most significant ones were observed after 24 h.

It can also be noted that all the films showed similar shapes of the curves, with the highest adsorption rate at the initial time (0–24 h), as a result of the high partial pressure of the saturated vapour, and without reaching equilibrium at the applied time (96 h). This behaviour indicates that the analysed films exhibit a hydrophilic nature, and the process connected with water adsorption was from monolayer adsorption to probably capillary adsorption. This is more evident, especially for control films (AP), whereas the films containing GDL showed similar final water contents (from 0.80 to 0.91 g/g d.m.), but control films were characterised by much higher water contents (1.45 g/g d.m.). Moreover, the greater the lactone addition, from 2.5 to 10%, the lower the amount of the water vapour adsorbed. The observed effect is explained by the modified film matrix structure, which is the effect of the presence of the GDL and its ability to affect the microscopic changes. This is probably due to the higher hydrophobic nature of the films. Therefore, it can be assumed that lower and higher contents of this aromatic compound did not show good miscibility and compatibility between the components.

Different tendencies in water vapour sorption kinetics as a result of the active compound addition to the biopolymer-based films have been observed previously. For example, the incorporation of phenolic acids to pectin films showed both higher and lower adsorption rates due to the type of the compound added [53]. Similar observations were also made for other active films based on pectin. Biratu et al. [54] observed a higher adsorption rate of water vapour for coffee pulp pectin films modified with the additions of glycerol, honey, and propolis. The authors observed that the moisture absorption of the analysed films initially increased to a peak at 24–48 h and then gradually decreased over time, reaching the equilibrium for all the samples at 216 h. Similar results were also observed for pectin films with the addition of pineapple juice [55].

### 3.7. The Effect of the GDL on the Water Vapour Sorption Isotherms of the Pectin-Based Packaging Films

**Figure 5:**
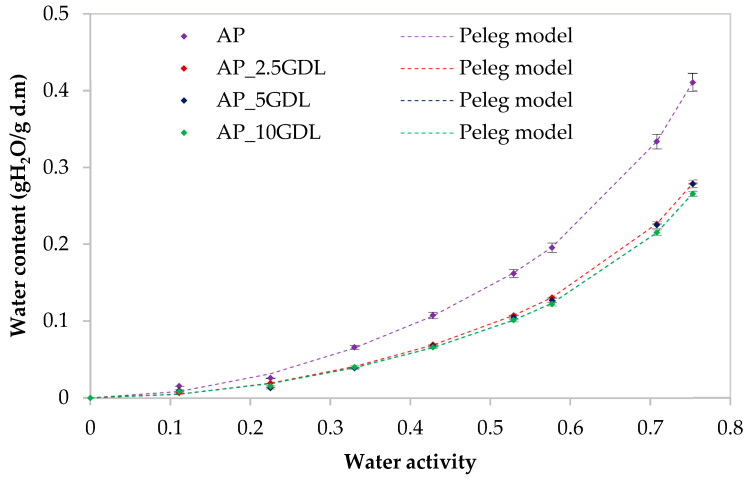
Fitting the Peleg model to describe the sorption isotherms of the apple pectin (AP) films containing GDL.

**Figure 6:**
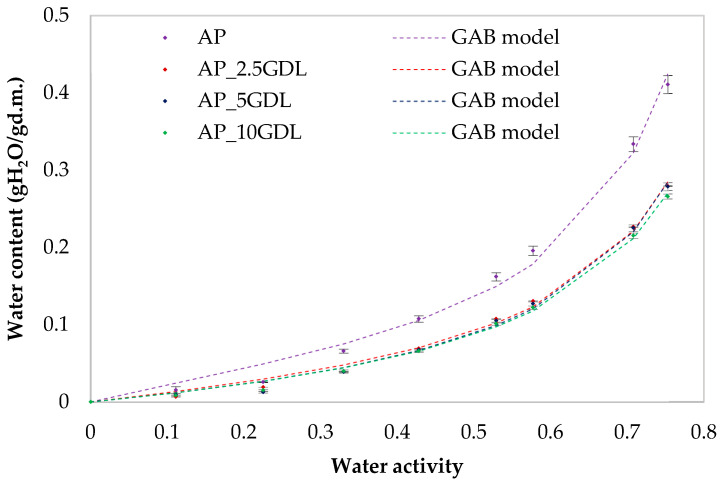
Fitting the GAB model to describe the sorption isotherms of the apple pectin (AP) films containing GDL.

The application of innovative films as food-packaging materials is still limited because of, for example, hydrophilicity, which affects the films’ properties [56,57,58]. That is why the determination of the sorption isotherms, which describe the relationship between the water content and water activity in a given material, is very important for the design, modelling, and optimisation of the drying, storage, and transportation of such materials [56]. For apple pectin (AP) films containing GDL, sorption isotherms in the water activity range 0–0.35 attained lower values of the equilibrium moisture content, while after exceeding 0.35, it increased rapidly (Figure 5 and Figure 6). The control sample (AP) shows the highest sorption dynamics, while the addition of the GDL significantly decreased the sorption dynamics, reducing hygroscopicity at all the water activity ranges. At each relative humidity, the equilibrium moisture content of the pectin films decreased with increasing GDL concentration from 2.5 to 10%. Apple pectin films can have an uptake of up to 41% moisture when conditioned at a water activity of 75.3% RH and 25 °C, while samples with GDL have only ~28%. The sorption isotherms of the pectin films with GDL can be classified as type III isotherms according to the Brunauer et al. [59] classification.

Ngo et al. [40] (for active edible films based on pectin and nanochitosan) and Todisco et al. [60] (for edible coatings based on pectin and guava byproducts) obtained sorption isotherms of type II, which is typical for pectin. Whereas Vera et al. [56] (for pectin–propolis films) obtained type III sorption isotherms. The authors explained that it is important to consider that all the studies had a different pectin source and different additives, which may change the isotherm type. The type III behaviour indicates that the main components of the product have little affinity for water molecules [40,60,61]. At low and medium values of water activity, the moisture content increased linearly with increasing water activity, while at high values of water activity, the water content increased rapidly, which was associated with capillary condensation. The product will absorb water at room temperature and a relative humidity of >35. Storage with a water activity of <0.35 makes the product be more stable. Saberi et al. [62] explained that at lower relative humidities, water is strongly adsorbed to the binding parts of the film’s surface, but with the increase in the water content, as a result of the swelling of the hydrophilic network of films, new sites for water were available to bind, increasing the moisture content.

Raharitsifa and Ratti [63] indicated that the chemical compositions and structures of food products may make it difficult to select the appropriate model describing the water vapour sorption curves. In Table 4 the variables of the individual models and the calculated fitting coefficients to describe the *RMS*, *MRE*, *SEE*, *R*^2^, and *RSS* sorption isotherms for each of them are presented. An MRE value of less than 10% indicates a correlation that provides a good representation of the data [64].

The Peleg and GAB models showed values of this index below 10%, and in the case of the Peleg model, these values were lower than those in the GAB model. It was found that the RMS values were low (for the control sample, ranging from 9.91% (Peleg’s model) to 21.04% (GAB’s model)). In the case of the samples with GDL (GDL), the lowest RMS coefficient occurred in the case of the Peleg model at the level of 5.65% and at 16.32% for the GAB model. A similar distribution of results was observed for the MRE, RSS, and SEE coefficients, where the Peleg model showed the lowest values. The lower the calculated MRE and SEE values, the better the model’s ability to represent the experimental data [31]. The residual sum of squares (RSS) is important in the nonlinear regression process, and the fitting procedure is designed to achieve the minimum value [65]. The values of the coefficients of determination (R^2^) for the Peleg model were 0.999, and for the GAB model, 0.991. To sum up all the results, the Peleg model is the best-fitting model for describing the sorption isotherms of all the samples. The fit of this model to the description of the obtained sorption isotherms is shown in Figure 5.

### 3.8. The Effect of the GDL on the Water Contact Angle of the Pectin-Based Packaging Films

**Figure 7:**
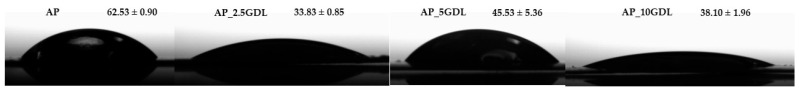
Water contact angles of the apple pectin (AP) films with GDL. The contact angle values (°) measured at 0 s are displayed directly on each image.

Surface hydrophobicity is a key parameter that determines the sensitivity of the film to water or moisture. It is most often assessed based on the measurement of the wetting angle between the film surface and the applied water droplet [66]. The analysis of the images (Figure 7) and data from Table 5, presenting measurements of the water’s wetting angles on edible films based on apple pectin with the addition of GDL at various concentrations (2.5–10%), allows for the assessment of the effect of this compound on the hydrophobicity of the material surface. To facilitate a visual comparison, the numerical contact angle values (measured at 0 s) have been overlaid directly on each droplet image in Figure 7.

The control films (AP) were characterised by the highest contact angles of 62.53° in air and 64.03° on the support, confirming the moderate hydrophobic nature of the surface. With time, these values gradually decreased to about 44°, indicating the slow spreading of the water droplets and the partial absorption of the material. In general, higher contact angle values correspond to higher surface hydrophobicity, with a higher ability of the surface to repel water [32].

The use of the GDL at a concentration of 2.5% resulted in significant reductions in the contact angle already in the first measurement—to 33.83° (air) and 38.35° (support), and within 30 s these values dropped below 30°, which indicates a significant increase in wettability and a more hydrophilic nature of the surface. This effect may be related to the presence of polar groups and their interactions with water, as well as to the potential reorganisation of the surface structure of the film [67]. In the case of the film containing 5% GDL, renewed increases in the wetting angle to 45.53° and 38.60° were observed, which may be due to the formation of a more compact, less accessible-to-water surface layer and the favourable distribution of the additive in the polymer matrix [51]. The highest concentration of GDL (10%) caused a renewed decrease in the wetting angle (to approximately 38°), which may be the effect of the supersaturation of the structure with the additive, leading to surface disturbances. A study by Azizah et al. [68] on gelatine–pectin films with the addition of lemongrass oil confirmed that hydrophobic additives can affect the increase in the wetting angles of the films, improving their resistance to water.

### 3.9. The Effects of the GDL on the Thermal Properties of the Pectin-Based Packaging Films

**Figure 8:**
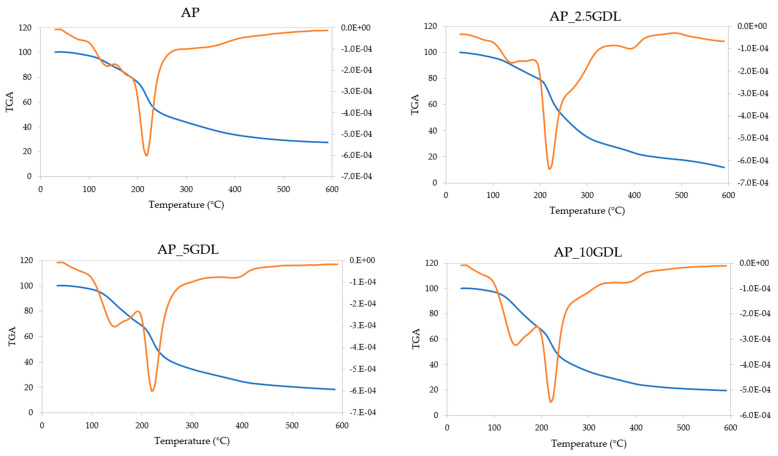
Thermogravimetric analysis (TGA) (blue) and derivative thermogravimetry (dTG) (orange) curves of the tested apple pectin (AP) films with GDL (GDL) added.

The pectin films with the addition of the GDL were investigated regarding their thermal properties. Thermogravimetric analysis (TGA), which allows the evaluation of the material’s thermal decomposition process when the material is subjected to linear heating in a nitrogen atmosphere, was performed. The results are presented in Figure 8, where the weight loss kinetics are presented as a function of the temperature (TGA curves—blue) and their first derivatives (dTGA—orange), from which the peak temperature of the decomposition was determined (Table 6). The course of the obtained curves exhibits a similar pattern, thus indicating the comparable stabilities of all the films upon exposure to elevated temperatures. However, the film containing 2.5% GDL (AP_2.5GDL) displayed moderately different properties from the others.

Four main stages of the films’ disintegration were recognised. The first stage occurred at temperatures from 30 to 95 °C, resulting in a weight loss of 2.15–3.35%, representing mostly water evaporation. The lowest change was observed for the sample with the highest content of GDL, while a remarkably higher weight loss was observed for the sample with the 2.5% addition. This indicates that the films consisted mostly of solid matter with a low content of water and possibly volatile compounds, which can also evaporate when the temperature rises [69].

The second stage was notably affected by the concentration of the GDL. Increasing the incorporation of the additive resulted in elevated stability, as portrayed by the elevated peak temperature starting from 131.45 °C and increasing by 12 °C. Compared to the control sample, the sample with the introduction of the GDL at the lowest level resulted in a slight reduction in the weight loss. However, the films with a greater amount of GDL showed the opposite tendency of increased weight loss from 18.78 to 25.96 and 27.45%.

The third stage of the decomposition occurred within the same range of temperatures, including the peak temperature (217.45–220.64 °C), in all the tested samples. The results observed for the control film and those with 5 and 10% additions of GDL were similar, while the sample containing 2.5% of the compound exhibited a remarkably higher weight loss (53.65%).

During the fourth stage of the thermal disintegration, the highest variety between the obtained results was observed. The temperature of the decomposition was not clearly defined for the control sample, while the samples incorporated with 2.5 and 5% GDL degraded mostly at 394–395 °C, and for the sample with the addition at the highest level, the peak decomposition temperature was 10 °C lower. During that last stage, the recorded weight loss seemed to increase after the addition of the GDL. However, its increasing concentration was followed by a reduction in the amount of components disintegrating at temperatures between 360 and 600 °C.

As stated by Lupaşcu et al. [70], the thermal depolymerisation and decomposition of apple pectin occur between 180 and 270 °C, which is followed by the decomposition of more stable compounds present in the composite matrix. The second peak of the increased decomposition rate appears at around 450 °C. The removal of the glycerol also occurs at approximately 260 °C, which is its evaporation temperature [71]. According to Ramos-Durán et al. [72], the thermostability of lactones and their blends highly depends on the chemical structures and the ratio of the selected compounds. They found that different types of lactones are usually stable up to 200 °C, and then the peak temperature of the decomposition may vary in the range from 260 to 400 °C. Those findings also correspond to the thermal degradation rate of the ricinoleic acid, which is the main substrate used for biotransformation to GDL [11]. Although studies on different adsorbents used for ricinoleic acid recovery showed that this compound is prone to cross-linking interactions with the adsorbent material, it had no effect on its thermostability [73]. However, the fact that GDL is often applied as a flavour- and odour-modifying food additive, which locates it also in the group of volatile compounds, it needs to be considered as well [11]. Given the above, the evident distinction of sample AP_2.5GDL, featuring the lowest tested concentration of the GDL, while increasing its amount led to higher contents of components degrading at 95–190 °C, may be explained by the interactions between the components of the films. This may suggest that at this concentration (2.5%), GDL was fully enclosed and protected by the pectin matrix. Therefore, its degradation occurred simultaneously with the degradation of the polymer (mostly during the third stage). On the other hand, samples fortified at a greater level were not able to fully enclose this component, and that contributed to the earlier evaporation of the unbound portion, which is related to the tendency observed during the second stage of heating.

### 3.10. The Effects of the GDL on the Antioxidant Properties of the Pectin-Based Packaging Films

The use of edible films containing compounds with antioxidant properties can effectively limit oxidation processes in food products, which contributes to extending their shelf lives and maintaining quality. The introduction of materials with antioxidant properties also allows for limiting the use of synthetic preservatives or antioxidants, which may have adverse effects on consumers’ health [74]. The antioxidant activity of the apple pectin films, both with and without added GDL, is presented in Table 7.

The analysis of the results showed that the control sample (AP, without added GDL) was characterised by the highest antioxidant activity in both the DPPH test (66.24%) and the ABTS test (93.88%). The introduction of the GDL to the films caused a statistically significant decrease in the antioxidant activity in both tests, with this effect being more pronounced in the DPPH test. In the samples with added GDL (2.5%, 5%, and 10%), the DPPH activity was significantly lower and amounted to 37.29%, 36.21%, and 31.92%, respectively, without significant statistical differences between these groups. In the ABTS test, a decrease in antioxidant activity was also noted with an increase in the concentration of the GDL—the values were 73.42% for AP_2.5GDL, 81.78% for AP_5GDL, and 69.34% for AP_10GDL, where the samples with GDL differed significantly from the control sample.

The conducted studies showed that the addition of the GDL (GDL) to the apple pectin films did not increase, but actually decreased, their antioxidant activities, which is confirmed by the results of the DPPH and ABTS tests. This may be due to the low ability of GDL to neutralise free radicals or its unfavourable interaction with the pectin matrix. For comparison, in the work of Vergel-Alfonso et al. [75], the use of anthocyanins in pectin and beeswax coatings significantly increased their antioxidant properties. These results indicate that the effectiveness of additives depends mainly on their chemical structure. Polyphenols, such as anthocyanins, are widely recognised as compounds with high antioxidant activity, as noted by Khoo et al. [76], whereas lactones, such as GDL, exhibit much weaker antioxidant properties [56]. Therefore, the effectiveness of edible-film additives depends largely on their chemical structure. The antioxidant efficiency of edible films depends on various factors, such as the nature and proportion of the antioxidant used, the way it interacts with other ingredients, the substances forming the film, and the processing conditions. Careful consideration of these factors is necessary to optimise the antioxidant activities of edible films and improve the shelf lives and qualities of food products [74].

### 3.11. The Effect of the GDL on the Chemical Structure of the Pectin-Based Packaging Films

**Figure 9:**
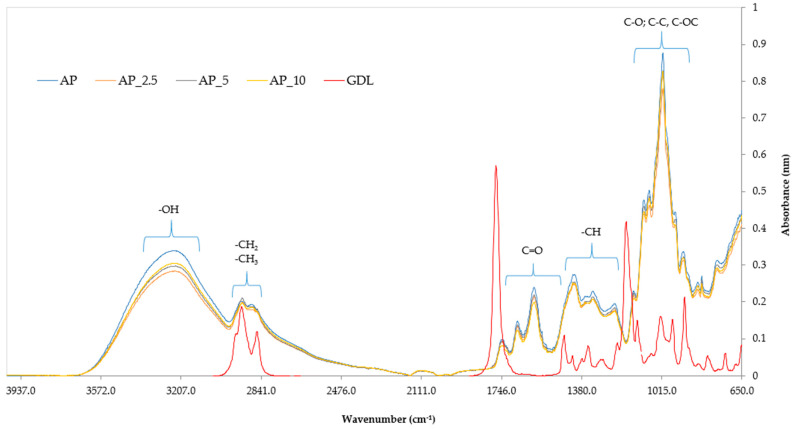
Fourier-transform infrared (FTIR) spectra of the pectin films (AP) with GDL and of the pure GDL.

Fourier-transform infrared spectroscopy (FTIR) is a technique that records the absorption of the electromagnetic radiation of specific wavenumber by oscillating molecules. When these molecules absorb energy, the oscillating atoms within their chemical bonds gain energy and transition to an excited state. This absorption process is represented in the form of an infrared spectrum, as noted by Enders et al. [77]. FTIR analysis is instrumental in identifying the functional groups present in a material’s structure. The characteristic infrared absorptions associated with various chemical bonds are analysed, allowing for the identification of specific functional groups. Each functional group has distinct absorption peaks, which facilitate the precise identification of the chemical components within a sample [78]. This method provides invaluable insights into the structural and chemical properties of the pectin films and pure GDL [78]. The spectra of the pectin films and pure GDL are shown in Figure 9.

The characteristic absorption peaks observed in the spectra are linked to various functional groups associated with specific chemical bonds: –OH, –CH, –CH_2_, –CH_3_, C=O, C–O, C–C, and C–OC. Within the electromagnetic wavenumber range 4000–3100 cm^−1^, vibrations corresponding to water molecules (H_2_O) become detectable, particularly pronounced in the control pectin sample (AP). Following this, in the range 3100–2800 cm^−1^, distinct vibrations from alkyl groups –CH_2_ and –CH_3_ are evident. Additionally, vibrations occurring between 1800 and 1500 cm−^1^ indicate the presence of carbonyl groups (C=O), which are typically indicative of aldehydes, esters, and aromatic acids. Moreover, a significant band at around 1500–1190 cm^−1^ supports the identification of methine groups (–CH). Lastly, in the spectral range 1900–900 cm^−1^, vibrations linked to C–O, C–C, and C–OC bonds reveal the existence of various saccharide groups, including arabinose, galactose, and glucose. Collectively, these findings offer essential insights into the chemical compositions of the analysed samples.

When comparing the control films and those with the added GDL, no significant differences were observed in the presence of functional groups or their absorption peaks. The pattern of the obtained spectra was similar to that of the spectra obtained for pectin films analysed by other researchers [79]. However, the analysis revealed significant differences between the spectrum of the pure GDL and the spectra of the pectin-based films. These findings suggest that GDL may undergo hydrolysis in the film-forming solution, which can be interpreted as the absence of certain functional groups in the film structure. This hydrolysis process suggests possible chemical changes occurring in the molecular structure of the GDL within the biopolymer matrix of the films [80].

### 3.12. The Effects of the GDL on the Antimicrobial Properties of the Pectin-Based Packaging Films

#### Evaluation of the Effect of the GDL on the Growth of Selected Species of Microorganisms in Pectin-Based Packaging Films

In assessing the validity of using GDL additives for edible coatings, the physicochemical parameters of a given film and its activity against microorganisms play essential roles. The idea of adding GDL to coatings was born from the literature-documented antimicrobial activities of compounds from the lactone group [43,44]. By adding GDL to edible films, it was assumed that a synergistic effect of the physicochemical and microbiological barrier would be achieved for products coated with it.

The antimicrobial activities of the coatings were assessed against three types of microorganisms: bacteria—*Bacillus subtilis*, yeasts, *Yarrowia lipolytica*, and fungi, *Monilinia fructicola*. The genera *Bacillus* and *Yarrowia* were chosen due to their wide distribution in nature. These microorganisms are isolated from soil, fresh and salt waters, and from animals’ digestive tracts or food. In addition, these species are characterised by a high growth rate and have the status of GRAS (generally recognised as safe) [45]. *M. fructicola* is the most destructive post-harvest microorganism of stone fruits, including apricots and peaches. It was decided to analyse the inhibitory effect of the GDL-containing films on this microorganism to show whether it is worth trying to cover apricots and peaches with such a coating to protect them from the development of this fungus during fruit storage, thus reducing the use of fungicides [46]. The use of the GDL in coating these fruits would also have an impact on improving the aroma because GDL is a natural, native compound with a creme–peach aroma [47]. Table 8 presents the results concerning the yields of the bacterial and yeast biomasses grown in media with the addition of a coating containing various concentrations of GDL.

The biomass yield results in the table above indicate that adding a lactone-containing coating to the culture medium inhibits microorganism growth. Media with the addition of coatings with a lactone concentration of 2.5% were characterised by an almost two-times-lower cell biomass yield than the control medium (containing a coating without the lactone), regardless of the tested microorganism species. In the case of *B. subtilis* bacteria, complete cell inhibition was already observed in media with the addition of 5% GDL. These results are consistent with the cell growth viability data collected in Table 9. *B. subtilis* cultures made from media with concentrations of 5% lactone or higher did not produce colony growth. In the case of the yeast *Y. lipolytica*, cell growth inhibition occurred above a 5% lactone concentration. Colonies were present on the tested medium with a 5% addition of GDL, with their concentration being 3 (after 48 h of culture) or 3.5 (after 72 h of culture) logarithmic units lower than that of the control medium (medium with a coating without the lactone).

The results presented in Table 8 and Table 9 consistently show that the number of *B. subtilis* cells in the medium with the addition of a lactone-free film is higher compared to those of the media without the film—the biomass yield is higher by about 1.5 g d.w./dm^3^, and the number of cells is higher by about one logarithmic unit. This suggests that the pectin film is a nutrient for these microorganisms. Pectin belongs to polysaccharides; it is a heteropolymer of a high molecular weight with a high content of galacturonic acid—an oxidised form of *D*-galactose, which is the central monomeric unit (about 65%) of the pectin molecule [48]. This polysaccharide can be degraded by enzymatic hydrolysis, with the participation of enzymes from the amylase class (EC 3.2.1). A special role in pectin hydrolysis is attributed to polygalacturonases (EC 3.2.1.15) [49].

Previous studies involving bacteria of the *Bacillus* genus indicate their production of amylolytic enzymes. The literature suggests that commercially available amylase preparations are produced mainly by the bacteria *Bacillus amyloliquefaciens*, *Bacillus stearothermophilus*, *Bacillus licheniformis*, and *Bacillus subtilis* [46]. This information allows us to conclude that pectin introduced to the *B. subtilis* culture medium is hydrolysed by these microorganisms and used as a carbon source, which results in more intensive microbial growth.

The pectin coating with the addition of the GDL also showed an inhibitory effect on the growth of the mycelium of *M. fructicola* (Table 10). The development of the mycelium of the pathogen was not completely inhibited at any tested concentration of GDL. Still, at 5% and 10% additions of GDL, it was reduced by almost 70% and 85%, respectively.

The results of this work show that GDL, a component of the packaging films, has an inhibiting effect on microorganisms of various species. This confirms the research conducted by Feron et al. [81], who indicated that GDL produced by microorganisms, after reaching a particular concentration in the medium, inhibits the growth of microbial cells. Aguero et al. [82], examining the basis of the mechanism of the peach aroma toxicity in yeast cells, indicated that lactones might inhibit the activity of H^+^-ATPase of microbial cells, resulting in a change in the integrity of the cell membrane. It is also probable that hydrophobic interactions are formed with the acyl chains of phospholipids of the cell membrane, leading to increased permeability disorders of the membrane. According to the authors, the intracellular pH of the cells also decreases, which results in their accelerated death.

Among the tested microorganisms, the lowest inhibition effect of the GDL coating was observed in the case of the yeast *Y. lipolytica*. This is probably due to their natural ability to synthesise this compound from fatty acids in the β-oxidation cycle. It is also assumed that the lower inhibitory effect compared to those of the other species may result, among others, from the fact that the cells of these yeasts synthesise surface-active compounds (surfactants). *Y. lipolytica* is known for the productions of Yansan [83] and liposan [84], which lead to the hydrophobisation of the cell surface [85] and, consequently, may, to some extent, limit the effect of the lactone dissolved in the microbiological medium on the cells.

## 4. Conclusions

Based on the conducted studies, it was found that the addition of the GDL significantly affects the optical, mechanical, and structural properties of pectin-based packaging films. The presence of the GDL reduced the film lightness (L*) and increased the colour saturation in the a* and b* spaces, which resulted in more intense shades of red and yellow. At the same time, the opacity of the material increased, which improved its barrier against UV–vis radiation, which may contribute to better protection of food products against photooxidation. The analysis of the mechanical properties showed that the introduction of the GDL caused decreases in the tensile strength and Young’s modulus while increasing the relative elongation of the film. These effects were particularly visible at higher concentrations of the compound. Structural observations revealed the formation of a porous film surface, and FTIR studies confirmed the lack of characteristic bands for pure GDL, which may indicate its partial hydrolysis in the film-forming solution. The films prepared with the additions of 2.5 and 5% were characterised by homogeneous structures and favourable mechanical properties, while at a concentration of 10 mL/100 mL, delamination of the material and migration of the compound were observed, which limit their potential applications.

Auspicious is the use of the GDL as a natural ingredient, improving the barrier and protective properties of the film while simultaneously inhibiting microorganisms of various species. The results obtained at this research stage suggest the idea of producing an innovative edible coating from GDL, which can be applied to perishable fruits (e.g., strawberries, apricots, and peaches) in which GDL is a natural aromatic component. This would allow the creation of a protective barrier for the fruit against physicochemical and microbiological changes occurring during storage while intensifying the fruit’s aroma. From a practical point of view, the modification of biopolymer films with GDL can be an effective tool for adjusting their functionality, depending on the application. The use of GDL as a natural component, improving the barrier and protective properties of the film while maintaining an appropriate level of the additive, is particularly promising.

## Figures and Tables

**Table 1:** The L*, a*, and b* colour parameters; total colour difference (Δ*E*); and opacity of the apple pectin (AP) films with the addition of the GDL.

Sample	L*	a*	b*	Δ*E*	Opacity (a.u./mm)
AP	83.68 ± 0.90 ^c^	−0.31 ± 0.24 ^a^	20.91 ± 2.05 ^a^	21.29 ± 2.24 ^a^	1.10 ± 0.09 ^a^
AP_2.5GDL	75.94 ± 0.98 ^b^	5.06 ± 0.59 ^b^	28.64 ± 0.66 ^b^	32.15 ± 1.14 ^b^	6.47 ± 0.69 ^b^
AP_5GDL	74.47 ± 1.07 ^a^	6.32 ± 0.78 ^c^	32.11 ± 0.35 ^d^	36.07 ± 0.83 ^d^	7.73 ± 1.02 ^bc^
AP_10GDL	76.10 ± 1.02 ^b^	5.82 ± 0.71 ^c^	30.77 ± 0.44 ^c^	34.05 ± 0.84 ^c^	8.64 ± 0.94 ^c^

Values are presented as means ± standard deviations. The same letters (^a–d^) indicate no statistically significant differences (*p* ≤ 0.05).

**Table 2:** The tensile strengths, elongations at break, and Young’s moduli of the apple pectin (AP) films with added GDL.

Sample	Tensile Strength (MPa)	Elongation at Break (%)	Young’s Modulus (MPa)
AP	13.84 ± 1.47 ^b^	14.00 ± 1.90 ^a^	108.34 ± 11.57 ^b^
AP_2.5GDL	4.31 ± 0.81 ^a^	11.50 ± 2.15 ^a^	44.91 ± 6.86 ^a^
AP_5GDL	5.98 ± 1.39 ^a^	21.26 ± 1.14 ^b^	50.87 ± 6.66 ^a^
AP_10GDL	5.68 ± 1.61 ^a^	19.97 ± 3.33 ^b^	46.12 ± 4.41 ^a^

Values are presented as means ± standard deviations. The same letters (^a–b^) indicate no statistically significant differences (*p* ≤ 0.05).

**Table 3:** Barrier properties of the apple pectin (AP) films with the added GDL.

Sample	Water Vapour Permeability (×10^−11^ g/m·s·Pa)	Oxygen Permeability (×10^−16^ g/m·s·Pa)	Carbon Dioxide Permeability (×10^−16^ g/m·s·Pa)
AP	1.97 ± 3.17 ^a^	1.49 ± 0.05 ^a^	4.47± 2.18 ^a^
AP_2.5GDL	2.49 ± 4.51 ^ab^	5.65 ± 0.09 ^ab^	8.03 ± 1.18 ^ab^
AP_5GDL	2.89 ± 2.25 ^ab^	12.06 ± 0.30 ^ab^	11.28 ± 0.77 ^ab^
AP_10GDL	3.23 ± 3.92 ^b^	15.19 ± 6.26 ^b^	12.74 ± 4.29 ^b^

Values are presented as means ± standard deviations. The same letters (^a–b^) indicate no statistically significant differences (*p* ≤ 0.05).

**Table 4:** Estimated parameter values (*A*, *B*, *C*, *u_m_*, *C*, and *k*), mean relative error (*MRE*), standard error of estimation (*SEE*), and residual sum of squares (*RSS*) of selected adsorption models.

Parameter	Unit	PELEG (a_w_ = 0.111–0.753)	GAB (a_w_ = 0.111–0.753)	Parameter	Unit	PELEG (a_w_ = 0.111–0.753)	GAB (a_w_ = 0.111–0.753)
		AP				AP_2.5GDL	
*u_m_*		-	0.0799	*u_m_*		-	0.0659
*A*		0.5286	-	*A*		0.3666	-
*B*		1.8940	-	*B*		1.9814	-
*C*		1.2848	2.6339	*C*		0.7771	1.6524
*D*		8.940	-	*D*		8.4687	
*K*		-	1.0962	*K*			1.0609
*S*	m^2^/g d.m.	-	284	*S*	m^2^/g d.m.	-	234
*RMS*	%	9.91	21.04	*RMS*	%	5.65	22.91
*MRE*	%	2.65	6.96	*MRE*	%	1.28	7.22
*SEE*		0.002	0.008	*SEE*		0.000	0.004
*RSS*		8.57 × 10^−5^	1.50 × 10^−3^	*RSS*		8.57 × 10^−5^	3.76 × 10^−4^
R^2^		0.999	0.991	R^2^		0.999	0.993
		AP_5GDL				AP_10GDL	
*u_m_*		-	0.0699	*u_m_*		-	0.0699
*A*		0.4192	-	*A*		0.3328	-
*B*		2.1882		*B*		1.9314	
*C*		1.1393	1.3250	*C*		0.7788	1.3416
*D*		10.771		*D*		8.3080	
*K*		-	1.0546	*K*			1.0424
*S*	m^2^/g d.m.	-	248	*S*	m^2^/g d.m.	-	248
*RMS*	%	9.37	21.57	*RMS*	%	9.37	16.32
*MRE*	%	2.58	5.62	*MRE*	%	2.58	5.04
*SEE*		0.001	0.003	*SEE*		0.001	0.003
*RSS*		2.69 × 10^−5^	3.76 × 10^−4^	*RSS*		2.69 × 10^−5^	2.32 × 10^−4^
R^2^		0.999	0.994	R^2^		0.999	0.994

**Table 5:** Water contact angles of the apple pectin (AP) films with added GDL.

Film	Film Side		Water Contact Angle (°)	
			Time (s)	
		0 s	15 s	30 s
AP	Air	62.53 ± 0.90 ^d^	45.45 ± 1.13 ^b^	44.05 ± 1.28 ^b^
	Support	64.03 ± 1.79 ^d^	45.53 ± 0.48 ^b^	43.98 ± 0.71 ^b^
AP_2.5GDL	Air	33.83 ± 0.85 ^a^	30.33 ± 3.42 ^a^	28.67 ± 3.06 ^a^
	Support	38.35 ± 0.62 ^ab^	29.15 ± 1.11 ^a^	27.45 ± 0.99 ^a^
AP_5GDL	Air	45.53 ± 5.36 ^c^	nd	nd
	Support	38.60 ± 0.71 ^ab^	nd	nd
AP_10GDL	Air	38.10 ± 1.96 ^ab^	nd	nd
	Support	39.18 ± 1.13 ^b^	nd	nd

Values are presented as means ± standard deviations. Identical letters (^a–d^) indicate no statistically significant differences (*p* ≤ 0.05); nd—not determined.

**Table 6:** Stages of thermal decomposition of the apple pectin (AP) films with added GDL.

Sample	1st Stage 30–95 °C		2nd Stage 95–190 °C		3rd Stage 190–360 °C		4th Stage 360–600 °C	
	T (°C)	Weight Loss (%)	T (°C)	Weight Loss (%)	T (°C)	Weight Loss (%)	T (°C)	Weight Loss (%)
AP	-	2.30	131.45	18.78	217.45	41.75	-	9.78
AP_2.5GDL	-	3.35	135.30	15.50	219.38	53.65	395.03	16.29
AP_5GDL	-	2.33	142.68	25.96	220.59	43.14	394.10	10.40
AP_10GDL	-	2.15	143.44	27.45	220.64	42.15	386.85	9.02

**Table 7:** Antioxidant properties of the apple pectin (AP) films with added GDL.

Film	ABTS (% Inhibition)	DPPH (% Inhibition)
AP	93.88 ± 0.74 ^c^	66.24 ± 8.14 ^b^
AP_2.5GDL	73.42 ± 5.22 ^ab^	37.29 ± 4.21 ^a^
AP_5GDL	81.78 ± 0.65 ^b^	36.21 ± 2.49 ^a^
AP_10GDL	69.34 ± 5.10 ^a^	31.92 ± 10.30 ^a^

Values are presented as means ± standard deviations. Identical letters (^a–c^) indicate no statistically significant differences (*p* ≤ 0.05).

**Table 8:** Biomass yields of microbial cells growing on Mueller–Hinton (*Bacillus*) or YPG (*Yarrowia*) media with the addition of pectin films with a varied GDL concentration.

	Biomass Yield (g d.w./dm^3^)			
	* Bacillus subtilis *		* Yarrowia lipolytica *	
Type of Medium	48 h	72 h	48 h	72 h
Without film	4.56 ± 0.23	6.09 ± 0.14	12.34 ± 0.19	15.89 ± 0.65
AP	6.23 ± 0.11	7.55 ± 0.27	13.01 ± 0.23	15.56 ± 0.22
AP_2.5GDL	3.02 ± 0.45	3.87 ± 0.31	7.23 ± 0.12	8.33 ± 0.08
AP_5GDL	0	0	1.24 ± 0.05	2.54 ± 0.11
AP_10GDL	0	0	0	0

**Table 9:** Cell growth viability cultivated on media with film containing different concentrations of GDL.

	Viability Indicator (log CFU/mL)			
	* Bacillus subtilis *		* Yarrowia lipolytica *	
Type of Medium	48 h	72 h	48 h	72 h
Without film	6.81 ± 0.10	8.09 ± 0.17	7.09 ± 0.22	8.20 ± 0.05
AP	7.91 ± 0.21	9.17 ± 0.04	7,11 ± 0.09	8.00 ± 0.32
AP_2.5GDL	5.62 ± 0.05	7.13 ± 0.41	6.87 ± 0.19	6.92 ± 0.18
AP_5GDL	0	0	4.09 ± 0.27	4.44 ± 0.20
AP_10GDL	0	0	0	0

**Table 10:** Inhibition effect of pectin films with GDL on the development of *Monilinia fructicola* on plates.

Type of Medium	Diameter of *Monilinia fructicola* (mm)	Inhibition Rate (%)
Without film	44.50 ± 1.08	-
AP	42.98 ± 2.12	-
AP_2.5GDL	22.95 ± 0.82	46.6
AP_5GDL	13.21 ± 0.40	69.3
AP_10GDL	6.52 ± 0.33	84.8

## Data Availability

The original contributions presented in this study are included in the article. Further inquiries can be directed to the corresponding authors.

## Supplementary Materials

The following supporting information can be downloaded at https://www.mdpi.com/article/10.3390/ma18163831/s1: Table S1: Compositions of the raw materials used for the production of the pectin films.
