# Supplementary material for: The Effects of Gamma-Decalactone on the Physicochemical and Antimicrobial Properties of Pectin-Based Packaging Films
Source: Materials (Basel). 2025 Aug 15;18(16):3831. doi: 10.3390/ma18163831 (PMC12387539; doi:10.3390/ma18163831)
Supplement: Supplementary file 1 [file materials-18-03831-s001.zip › materials-3728626-supplementary.pdf]

Supplementary materials

Table S1. Composition of the raw materials used for the production of pectin films.

Film	Biopolymer and Concentration	Gamma-Decalactone Concentration (%)	Glycerol Concentration (% relative to pectin)	Tween80 (%)
**AP**	Apple pectin 5%	0	30	0.5
**AP_2.5GDL**		2.5	30	0.5
**AP_5GDL**		5	30	0.5
**AP_10GDL**		10	30	0.5
